# Three different pathways of IgM-antibody-dependent hemolysis are mainly regulated by complement

**DOI:** 10.3389/fimmu.2023.1114509

**Published:** 2023-02-02

**Authors:** Thilo Bartolmäs, Axel Pruß, Beate Mayer

**Affiliations:** Institute of Transfusion Medicine, Charité-Universitätsmedizin Berlin, Corporate Member of Freie Universität Berlin, Humboldt-Universität zu Berlin, and Berlin Institute of Health, Berlin, Germany

**Keywords:** eryptosis, AIHA, complement, DAT, cold agglutinin, hemolysis

## Abstract

Antibodies to red blood cells (RBCs) may hemolyze erythrocytes *via* Fc-mediated phagocytosis or complement-dependent. Complement activation on RBCs can be detected by C3d-direct antiglobulin test (DAT), which is the only test in immune hematology that directly targets complement. However, a positive DAT with anti-C3d cannot distinguish between C3b-mediated extravascular hemolysis, C5b-C9-mediated intravascular hemolysis and C5b-C8-mediated eryptosis. Furthermore, DAT is not suitable to estimate the strength of hemolysis. Autoimmune hemolytic anemia (AIHA) is a rare disease that is caused by autoantibodies to red blood cells that is divided in warm AIHA and in cold agglutinin disease (CAD). The causative antibodies in CAD and sometimes in warm AIHA are from the IgM class. Depending on strength of complement activation they can induce extravascular hemolysis, intravascular hemolysis and eryptosis. We studied the three types of hemolysis by use of sera from patients with CAD under various conditions. We found that additionally to the routinely applied C3d-DAT, indirect tests for complement activity (free hemoglobin and Annexin V-binding to phosphatidylserine-exposing RBCs) should be used to determine the portion of extravascular, intravascular and eryptotic hemolysis. Eryptotic hemolysis may have a significant share in clinical relevant CAD or IgM warm AIHA, which should be considered for successful treatment.

## Introduction

The complement system is part of the innate immune system that serves mainly the clearance of apoptotic cells and the pathogen elimination. It can be launched *via* three distinct pathways, named classical, lectin and alternative pathway (1) that converge on the formation of an enzyme complex capable to convert complement component C3 into active products C3a and C3b (2). C3b binds to the C3 convertase to form C5 convertase which cleaves C5 into C5a and C5b. C5b recruits complement components C6, C7, C8 and C9 which ultimately form the membrane-attack complex (MAC) (1).

The alternative pathway is permanently activated by spontaneous hydrolysis that converts C3 finally into C3a and C3b (tick-over). RBCs are susceptible to complement attacks. Complement activation on RBC surface is regulated by two GPI-anchored proteins: CD55 (decay accelerating factor, DAF) that increases the removal of the complement complex C3 convertase, and CD59 (membrane inhibitor of reactive lysis, MIRL) that inhibits C9 binding to C5b, C6, C7 and C8, hence inhibiting the formation of the MAC. Due to a mutation in the X-linked phosphatidylinositol glycan class A (PIG-A) gene and subsequent deficiency in glycosylphosphatidylinositol (GPI) anchor, these GPI-linked proteins are absent or minimally expressed on paroxysmal nocturnal hemoglobinuria (PNH) blood cells (3).

Antibodies to red blood cells (RBCs) may be either allogeneic or autologous. They usually belong either to the IgG class (e.g. anti-D, warm autoantibodies) or IgM class (isoagglutinins: Anti-A, Anti-B or cold autoantibodies). Most antibodies are capable to activate complement (IgM, IgG1, IgG3) *via* the classical pathway, however, strength of activation is different with hexameric IgM > pentameric IgM >> IgG3 > IgG1 >> IgG2. Some antibody (sub)classes do not activate complement *via* the classical pathway (IgG4, IgA) (4).

Autoimmune hemolytic anemias (AIHA) are rare diseases caused by autoantibodies (aabs) against RBCs. These disorders are diagnosed on the basis of evidence of hemolysis in combination with characteristic serological findings, especially the direct antiglobulin test (DAT). This test is used to detect antibodies on RBCs and to identify their immunoglobulin class. The majority of cases of AIHA are mediated by warm-reactive autoantibodies, i.e., antibodies displaying optimal reactivity with human RBC at 37°C and which are usually of the IgG immunoglobulin class (5). In contrast, autoantibodies in cold agglutinin disease (CAD) are usually from IgM class directed typically against I/i carbohydrate antigens. Their thermal amplitude, i.e. the highest temperature they agglutinate RBCs, is usually below 32°C and their maximal reactivity is at 4°C (6). AIHAs can be either idiopathic or secondary due to a variety of diseases like leukemia, systemic lupus erythematosus, lymphoproliferative disorders, infections (7).

The pathogenic role of autoantibodies depends on their immunoglobulin class, subclass, thermal amplitude, as well as affinity and efficiency in activating complement (8).

IgG aabs in warm AIHA usually activate complement to C3b, leading to Fc-mediated phagocytosis (mostly in spleen) or C3b-mediated phagocytosis (mostly in the liver). Complement degradation also plays a key role in complement regulation. C3b is converted into iC3b within 1 min and in about 10 min further degraded into C3d, which no longer fixes complement, nor does it serve as an opsonin (2). C3d is fixed stably to RBCs by a molecular binding and is thus a marker of earlier complement activation.

In contrast to the aforementioned so-called extravascular hemolysis, intravascular hemolysis requires complete complement activation until the membrane attack complex. This can usually only be achieved with IgM antibodies, although there are reports of IgG-mediated intravascular hemolysis.

A single IgM molecule is capable of activating C1q (9). C1 can be activated already below 15°C, however, C4 needs temperatures above 18°C for activation (10). The C2/C4 complex, which is important for C3 activation, is unstable at elevated temperatures. Additionally, a large number of activated C3 and C5 (about 90000 molecules) are required for lysis (10). This explains why most patients with CAD have only a chronic mild (extravascular) hemolysis, since the thermal amplitude of IgM aabs is often below 20°C and the complement system is not fully activated. On the other hand, CAD patients with thermal amplitudes above 30°C or patients with warm IgM-AIHA may have severe or even fatal intravascular hemolysis (11).

Whereas intravascular and extravascular hemolysis have been known for a long time a third form of hemolysis, eryptosis, has only recently been considered to be a significant contributor to AIHA when IgM is involved (12). Eryptosis, the suicidal death of RBCs, resembling the apoptosis of nucleated cells has been described to occur in a variety of diseases (13, 14). Its hallmark is the breakdown of phosphatidylserine (PS) -asymmetry upon Ca^2+^ influx into RBCs. Phosphatidylserine- exposure can be easily accessed by annexin V-binding (15). We gave evidence of eryptosis in 7 of 13 patients with IgM warm AIHA and all patients with significant CAD (12). We could further show that IgM-mediated eryptosis requires complement (16). Incubation with fresh serum from CAD patients can induce eryptosis in donor O RBCs. This can be abolished by serum heat inactivation (i.a.) or EDTA addition prior to incubation with O RBCs. Furthermore, eryptosis is also suppressed strongly by adding anti-C5 (Eculizumab ^®^) or partially with anti-C8 but is increased with anti-C9. Incubation of O RBCs with heat-inactivated patients serum and single complement factor-deficient sera (C5, C6, C7, C8 or C9-deficient) revealed that eryptosis (exposure of PS at the surface of RBCs) is increased gradually in the sublytic terminal complexes to reach a maximum at the C5b-8 stage (16). The significance of eryptosis to AIHA is not yet clear, since eryptotic cells are quickly removed from circulation by phagocytosis (15). Thus, eryptotic RBCs mostly have the same fate as C3b-opsonized RBCs, all these cells undergo extracellular hemolysis. However, since classical IgG- or C3b-mediated extravascular hemolysis or eryptosis require different therapies, a more thorough differentiation may be beneficial. Therefore, we used sera from three different patients with clinically relevant CAD to further investigate the influence of IgM aab on donor RBCs under different conditions (temperature, titer, complement concentration) looking for signs of intravascular hemolysis (free hemoglobin), eryptosis (Annexin V-binding to externalized PS) and extravascular hemolysis (using C3d as a surrogate for C3b). All three hemolysis pathways usually run in parallel under physiological conditions, but to different extents depending on titer, temperature and complement factor concentration.

## Materials and methods

### Samples and reagents

Serum and EDTA samples were from three patients with clinically relevant AIHA of cold type (12). Fresh O RBCs and serum from healthy blood donors were used in the incubation experiments and as controls and source of complement, respectively. To diminish individual variability, fresh serum from three donors were pooled before use. This study was approved by the local Ethics Committee with registration number EA2/058/12.

### Direct antiglobulin test (DAT), eluates, antibody titers and thermal amplitude

Routine diagnostic tests were performed with EDTA samples in our immunohematological reference laboratory as described before for monospecific DAT, eluates and thermal amplitude (17, 18). Antibody titers were performed at 4°C.

### Hemolysis experiments

A typical experiment was performed by incubation of 20 µl donor RBCs (blood group O) with a total of 400 µl of a 1:10 mixture of patient’s serum with fresh donor serum as a source of complement or, if applicable, heat-inactivated donor serum, as indicated. The proportion of patient’s serum to donor serum was 1:10 to insure similar complement concentrations in parallel experiments. Incubation time and temperature was generally 48 hours at 20°C. 48 h was found to be the optimal time for measuring eryptosis after induction with patients serum (12). In thermal amplitude experiments, cells were incubated 4 hours at the indicated temperature, reaction was stopped by addition of EDTA and samples stored 48 hours at 4°C. The supernatant was then separated for determination of free hemoglobin and cells were washed carefully three times at 37°C to avoid sheer stress as a cause of RBC damage.

### Free hemoglobin

300 µl of supernatant from the hemolysis experiment were mixed with 1,2 ml hemoglobin buffer (Hämoglobinreagenz, Bioanalytic GmbH, Freiburg Germany) according to the manufacturers recommendation. After 5 min. incubation at room temperature cyanohemoglobin was measured in a Hitachi photometer (wavelength 671 nm).

### Complement factor C3d on donor RBCs (C3d-DAT)

Twenty-five µl of anti-C3d (diluted 1:40, from DAKO, Agilent Technologies, St. Clara, USA) were added to 50 µl of warm washed O RBCs from hemolysis experiments and examined for agglutination using the buffered gel card (ID-card “NaCl, Enzyme Test and Cold Agglutinins” Bio-Rad, Cressier sur Morat, Switzerland) (18). As a control for autoagglutination RBCs were centrifuged without anti-C3d.

### Flow cytometry

Eryptosis was measured as previously described (12). Annexin V-binding to extracellular exposed PS was used as a marker of eryptosis. Briefly, 5 µl of washed RBCs from hemolysis experiments were diluted in 200 µl Annexin V-binding buffer (BD Biosciences, Heidelberg, Germany). Therefrom 5 µl of RBCs were added to 5 µl of PE-(phycoerythrin) Annexin V in 45 µl of Annexin V-binding buffer (BD Biosciences, Heidelberg, Germany). After incubation at room temperature for 15 min, the mixture was diluted with 450 µl of Annexin V-binding buffer and analysed by flow cytometry (MACSQuant^®^ Flow Cytometer, Miltenyi Biotech, Germany). At least 20 000 events were collected for each sample. Data were analyzed using the FlowJo^®^ software (FlowJo, LLC; USA) and the percentage of PE-Annexin V positive cells compared to the negative controls were calculated. A positive control was generated by incubation of 5 μL RBCs in 200 μL of a HEPES-buffered Ringer’s solution with the Ca^2+^ ionophore ionomycin (1 μmol L^−1^).

### Statistics

For each data point 2-5 experiments were performed. Data are expressed as arithmetic means ± standard error of the mean (SEM) and statistical analysis was performed using IBM SSPS statistics software v24. Significance between two groups (patient vs. negative control) was determined using unpaired student’s T test with probabilities of P < 0.05 considered statistically significant.

## Results

### DAT, eluates, antibody-titers and thermal amplitude

All three patients had a typical serological picture of a cold agglutinin disease. Monospecific DAT was strongly positive only with anti-C3d; with anti-IgM, anti-IgG, anti-IgA it was negative. Eluates were negative. In the serum, cold agglutinins with I-specificity were found with a thermal amplitude of 35° (P1), 28° (P2) and 22°C (P3), respectively. The aabs-titers found at 4°C were 1024 in P1 and P2, and 512 in P3.

### Intravascular hemolysis, extravascular hemolysis and eryptosis occur simultaneously

**Figure 1:**
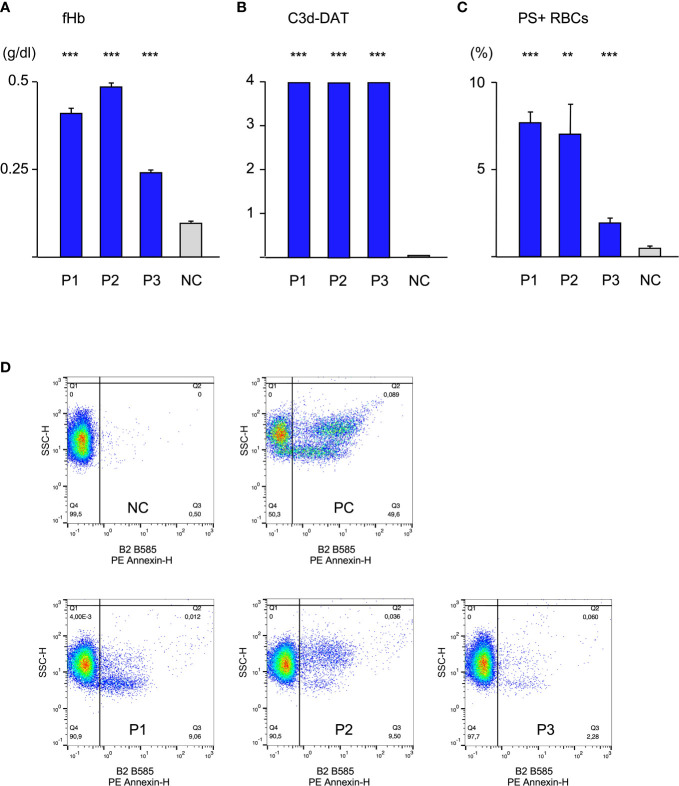
Hemolysis parameter from incubation of O RBCs with patients’ sera (dilution 1:10 with fresh AB serum, 48 hours at 20°C), **(A)** free Hemoglobin (fHb), **(B)** direct antiglobulin test with anti-C3d (C3d-DAT, reaction strength: 0 no agglutination, 1 weak, 2 moderate, 3 strong, 4 very strong), **(C)** Annexin V-binding to phosphatidylserine- exposing RBCs (PS+RBCs); arithmetic means ± SEM. Five replicate experiments have been performed. *** statistically highly significant (p < 0,001), ** very significant (p < 0,01) vs negative control. **(D)** Scatter plots showing PE Annexin V-binding to O RBCs after incubation with serum from Patients P1-P3, NC negative control (AB serum), PC positive control (incubation of RBCs with ionomycine).

Initially, patients’ sera were diluted with fresh donor serum (1:10), as a source of complement and added to donor cells at 20°C for 48 h. As a control RBCs were treated with donor serum. Experiments were performed in triplicate (**Figure 1**). RBCs treated with serum of either patient showed a strong C3d-load (positive C3d-DAT). Since C3d results from a quick degradation of C3b within approximately 11 minutes (2), it may be considered as a substitute for potential C3b-mediated extravascular hemolysis. However, it is not clear how large the share of ingested C3b-RBCs compared to C3d-RBCs is, since it may depend heavily on patients’ macrophage count and activity. C3d was not found on RBCs from control experiments. Similarly, free hemoglobin (fHb) was detectable in the supernatant of all experiments with patient sera, giving evidence of intravascular hemolysis. Serum of patient 3 generated less free hemoglobin, which corresponds to its lower thermal amplitude. Annexin V-binding to externalized phosphatidylserine (PS+ RBCs) as a marker for eryptosis was elevated in all patients’ experiments and again sera from patient 1 and 2 produced stronger eryptosis than serum from patient 3. These results show that all three forms of hemolysis occur simultaneously and the thermal amplitude may play a significant role for intravascular hemolysis and eryptosis.

### Thermal amplitude is of main significance for the type of hemolysis

**Figure 2:**
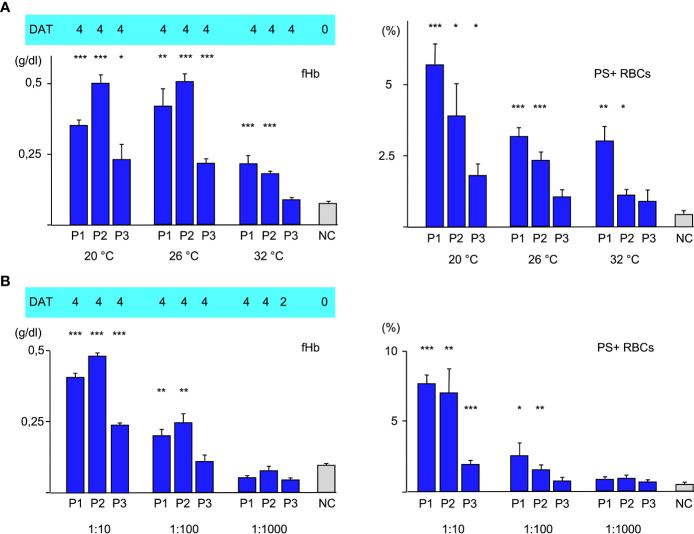
**(A)** Hemolysis parameter from incubation of O RBCs with patients’ sera (dilution 1:10 with fresh AB serum, 4 hours at different temperatures): top C3d-DAT (reaction strength: 0 no agglutination, 1 weak, 2 moderate, 3 strong, 4 very strong), left free Hemoglobin (fHb), right Annexin V-binding to phosphatidylserine- exposing RBCs (PS+RBCs). Three replicate experiments have been performed. *** statistically highly significant (p < 0,001), ** very significant (p < 0,01), * significant (p < 0,05) vs negative control. **(B)** Hemolysis parameter from incubation of O RBCs with patients’ sera (at different dilutions 1:10, 1:100, 1:1000 with fresh AB serum, 48 hours at 20°C): top C3d-DAT (reaction strength: 0 no agglutination, 1 weak, 2 moderate, 3 strong, 4 very strong), left free Hemoglobin (fHb), right Annexin V-binding to phosphatidylserine- exposing RBCs (PS+RBCs). Three replicate experiments have been performed.

To further elucidate the role of the thermal amplitude, we performed experiments at different temperatures (**Figure 2A**). Free hemoglobin was strongly increased at 20°C and 26°C in all cases but at 32°C it could only be detected to a much lesser extent with sera of patients 1 and of patient 2. Free hemoglobin was not elevated with serum from patient 3 at this temperature. Annexin V-binding was found to be highest in all cases at 20°C; at 26°C it was substantially lower but still elevated. At 32°C, eryptosis was only strongly detectable in experiment with serum from patient 1. C3d was bound strongly at all temperatures.

### Variation of the antibody concentration

In the next experiments, patients’ serum was used at different dilutions ranging from 1:10 to 1:1000 for incubation with O RBCs at room temperature (**Figure 2B**). Sera of all patients induced hemolysis, eryptosis and complement deposition at RBCs at a titer of 10. At a dilution of 1:100, free hemoglobin and Annexin V-binding were detected only with patients’ sera 1 and 2. At a dilution of 1:1000 only C3d-deposition was detected after incubation with all patients’ sera, indicating that at this dilution only extravascular hemolysis may take place.

### Complement variation

To analyze the effect of disposable complement on the three types of hemolysis, fresh serum as a source of complement was mixed with heat-inactivated serum at different ratios, added to patients’ serum 10:1 and used for incubation with O RBCs for 48 h at 20°C. As expected, free hemoglobin and Annexin V-binding to RBCs decreased with lower concentration of fresh serum, indicating decreasing intravascular hemolysis and eryptosis. C3d-binding was strong as shown by DAT. Unexpectedly, using more than 90% heat-inactivated serum with aab from P1, but not P2 and P3 led to a very strong agglutination of O RBCs and made measurement of PE-Annexin V in flow cytometry impossible, since it was difficult to separate the RBCs without damaging them to much.

## Discussion

Intravascular hemolysis and extravascular hemolysis due to alloantibodies or autoantibodies (aab) to RBCs are known since many years. Whereas the first is caused by the membrane attack complex (C5b-C8) induced mostly by IgM antibodies, the latter is either elicited by IgG antibodies alone or by complement component C3b that is bound to RBCs following antibody (IgG/IgM) contact. In contrast, eryptosis as a cause of aab-mediated hemolysis has been shown first a few years ago by our group (12). Lately, we found that sublytic terminal component complexes (especially C5b-C8) are responsible for IgM-mediated eryptosis (16). Interestingly, more than 40 years ago, independent groups found a C5b-C8 or C8-mediated hemolysis that differed in its mechanism from the already known intravascular hemolysis (19, 20). They found that C5b-C8 or C8-mediated hemolysis (like the later found eryptosis) was delayed for several hours, whereas intravascular hemolysis occurred instantaneously. In this work, we measured all three types of hemolysis during experiments *in vitro* using IgM-aab from patients with cold agglutinin disease.

Though our data is relatively small, due to the rarity of this disease, it still suggests that all three types of hemolysis may occur simultaneously. This is an important finding, since eryptotic cells are either rapidly removed from circulation by macrophages (extravascular hemolysis) or undergo membrane blebbing, cell shrinkage, membrane fragility and finally intravascular hemolysis (14). Thus, eryptotic cells in circulation are usually underestimated. In our *in vitro* experiments, eryptotic cells represent up to more than 10% of the O RBCs with patients’ sera diluted tenfold. However, since eryptosis may require specific therapy, as we have already shown by use of erythropoietin (12), it is recommendable to determine eryptotic cells in IgM warm autoimmune hemolytic anemia (AIHA) or cold agglutinin disease (CAD).

Moreover, our data confirm that thermal amplitude is the pivotal feature in patients with CAD as can be seen in **Figure 2A** in the difference between Patients 1 and 2 vs. 3. In case of patients 2 and 3, free hemoglobin as a marker of intravascular hemolysis and Annexin V-binding as a marker of eryptosis were detectable even above the thermal amplitude that was measured by agglutination. Patient 1 had a thermal amplitude of more than 32°C, therefore we cannot make a statement about this case. Another noticeable point is the fact that free hemoglobin remained pretty much unchanged comparing 20°C and 26°C, whereas eryptosis was substantially lower. This may be explained by the fact that the strength of complement system is dependent from temperature. A more activated complement system may promote intravascular hemolysis (C5b-C9, MAC) at the expense of eryptosis (C5b-C8) as long as the IgM aab are sufficiently bound. Agglutination of CAD is weakening with rising temperatures, therefore there is an oppositional effect of complement activation and antibody binding to RBCs. Likewise, the similar amount of PS-exposing RBCs at 26°C and 32°C in the experiment with serum from P1 may be explained by stronger complement activation compensating weaker aab binding. The C3d-load of RBCs did not change with temperature. The fact that IgM aab bind at least for a short time even at core-temperature (37°) has been shown before and was used as an explanation for the mild hemolysis that occurred in some patients even in hot summer months (21).

In the next set of experiments, we compared different antibody dilutions (**Figure 2B**). As expected, both, fHb and PS+RBCs declined with dilution of the aab. At a dilution of 1:1000 only C3d could be detected on the O RBCs. At this titer only very few IgM aab may attach to the RBCs, however it was shown before that one IgM molecule is suffient to bind one C1 molecule (9).

What seems more interesting, is that even in a 100 fold dilution of aab there is still a remarkable intravascular hemolysis and eryptosis detectable by using sera from patient 1 and 2. This may explain why even when the beneficial effect of rituximab (anti-CD20) has been shown in two recent prospective trials, the partial response rate was about 50% and only few complete remissions could be seen (22).

**Figure 3:**
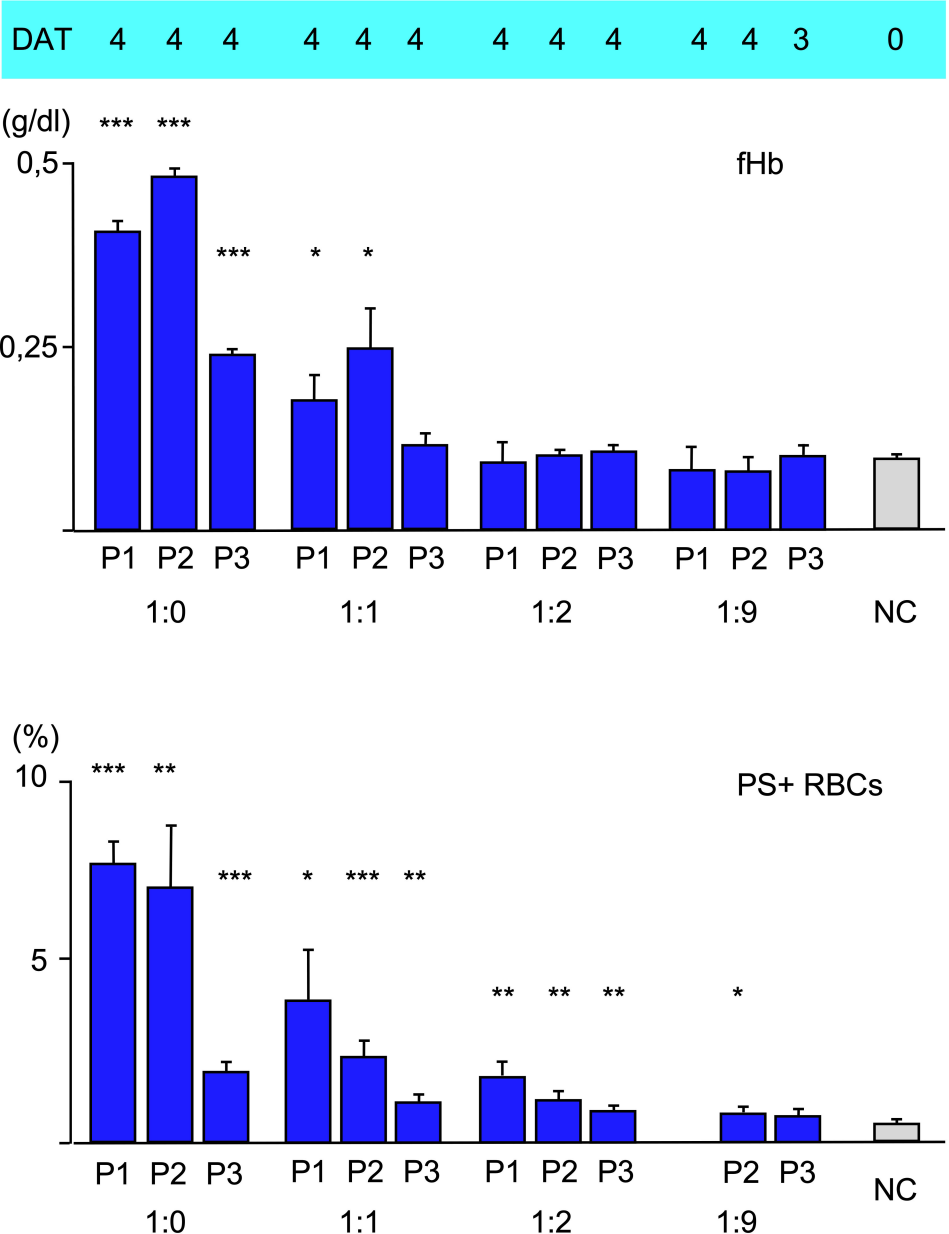
Hemolysis parameter from incubation of O RBCs with patients’ sera (dilution 1:10) with a mixture of fresh AB serum and heat-inactivated (i.a.) AB serum, 48 hours at 20°C): 1:0 only fresh AB-serum, 1:1 50% fresh, 50% i.a. AB serum, 1:2 34% fresh, 66% i.a. AB serum, 1:9 10% fresh, 90% i.a. AB serum. top C3d-DAT (reaction strength: 0 no agglutination, 1 weak, 2 moderate, 3 strong, 4 very strong), middle free Hemoglobin (fHb), bottom Annexin V-binding to phosphatidylserine- exposing RBCs (PS+RBCs). Three replicate experiments have been performed (with mixture 1:2 and 1:9: two replicate experiments). *** statistically highly significant (p < 0,001), ** very significant (p < 0,01), * significant (p < 0,05) vs negative control.

In a final experiment, the amount of fresh donor serum as a source of complement was varied (**Figure 3**). A replacement of 50% fresh serum with heat-inactivated serum seems to have a rather huge effect on free hemoglobin (intravascular hemolysis) and Annexin V-binding (eryptosis). This result hints to complement inhibition as an effective therapeutic option in clinical relevant CAD/AIHA as is currently done with the newly emerged C1s-antibody (sutimlimab^®^) (23). At 33% fresh serum complement (by dilution with heat-inactivated serum 1:2) or below only a C3d-positive DAT (extravascular hemolysis) is detectable.

As a conclusion, all three forms of hemolysis are detectable together in patients with clinically relevant cold agglutinin disease. In patients with a low thermal amplitude of 4°C only some extravascular hemolysis may occur, but the general importance of the thermal amplitude could be clearly shown, especially the fact that strong complement activation to sublytical complement complexes or the membrane attack complex can be seen above the thermal amplitude determined by agglutination. Therefore avoidance of exposure to the cold is the main therapeutic approach in these patients. Medical treatment is difficult, which is also shown in this work, by demonstrating a remarkable residual fHb and eryptosis at substantially lowered aab titers and substitutive effects of intravascular hemolysis by eryptosis in case of lowered complement factors.

However, an accommodating combination of rituximab, erythropoietin or C1s-antibody (sutimlimab^®^ (23) and in cases of severe life-threatening hemolysis C5-antibody (eculizumab^®^) may be beneficial in patients with difficult to control CAD.

Routine diagnosis in clinical relevant patients with cold agglutinin disease may be incomplete as it is done today. A positive C3d-DAT is valuable information to suspect IgM aab, however in patients with CAD as well as in our experiments reaction strength is mostly very high, without correlation to the clinical picture. Thermal amplitude may be the best routine parameter to estimate clinical relevance of cold agglutinins (21). Hemolysis parameter (lactat dehydrogenase, haptoglobin, bilirubin, hemoglobin) are mandatory and serum complement C3 and C4 levels may be helpful to assess a clinical picture (24). However, to get a better insight of IgM aab-induced complement activity and its caused types of hemolysis, our data indicate that free hemoglobin and eryptosis should be determined additionally.

Our findings on the three different types of immune hemolysis may apply beyond CAD, since eryptosis has been shown also for IgM-warm autoantibodies (12). In addition, we recently found strong eryptosis and intravascular hemolyis in a patient with sickle cell disease due to an irregular IgM alloantibody directed against the Lewis-Antigen Le(b) (unpublished data).

## Data availability statement

The raw data supporting the conclusions of this article will be made available by the authors, without undue reservation.

## Ethics statement

The studies involving human participants were reviewed and approved by Ethikkommission der Charité, Charitéplatz 1, 10117 Berlin, Vote No. EA2/058/12. Written informed consent for participation was not required for this study in accordance with the national legislation and the institutional requirements.

## Author contributions

TB designed and performed the experiments. BM treated the patients. TB, BM and AP analyzed the data. TB wrote the paper with input from all other authors. All authors contributed to the article and approved the submitted version.
